# Supplementary material for: Enhanced SREBP2-driven cholesterol biosynthesis by PKCλ/ι deficiency in intestinal epithelial cells promotes aggressive serrated tumorigenesis
Source: Nat Commun. 2023 Dec 13;14:8075. doi: 10.1038/s41467-023-43690-5 (PMC10719313; doi:10.1038/s41467-023-43690-5)
Supplement: Supplementary file 1 — Supplementary Information [file 41467_2023_43690_MOESM1_ESM.pdf]

SUPPLEMENTARY INFORMATION

Enhanced SREBP2-driven cholesterol biosynthesis by PKCλ/ι deficiency in intestinal epithelial cells promotes aggressive serrated tumorigenesis

Yu Muta, Juan F. Linares, Anxo Martinez-Ordoñez, Angeles Duran, Tania Cid-Diaz, Hiroto Kinoshita, Xiao Zhang, Qixiu Han, Yuki Nakanishi, Naoko Nakanishi, Thekla Cordes, Gurpreet K. Arora, Marc Ruiz-Martinez, Miguel Reina-Campos, Hiroaki Kasashima, Masakazu Yashiro, Kiyoshi Maeda, Ana Albaladejo-Gonzalez, Daniel Torres-Moreno, José García-Solano, Pablo Conesa-Zamora, Giorgio Inghirami, Christian M. Metallo, Timothy F. Osborne, Maria T. Diaz-Meco, and Jorge Moscat

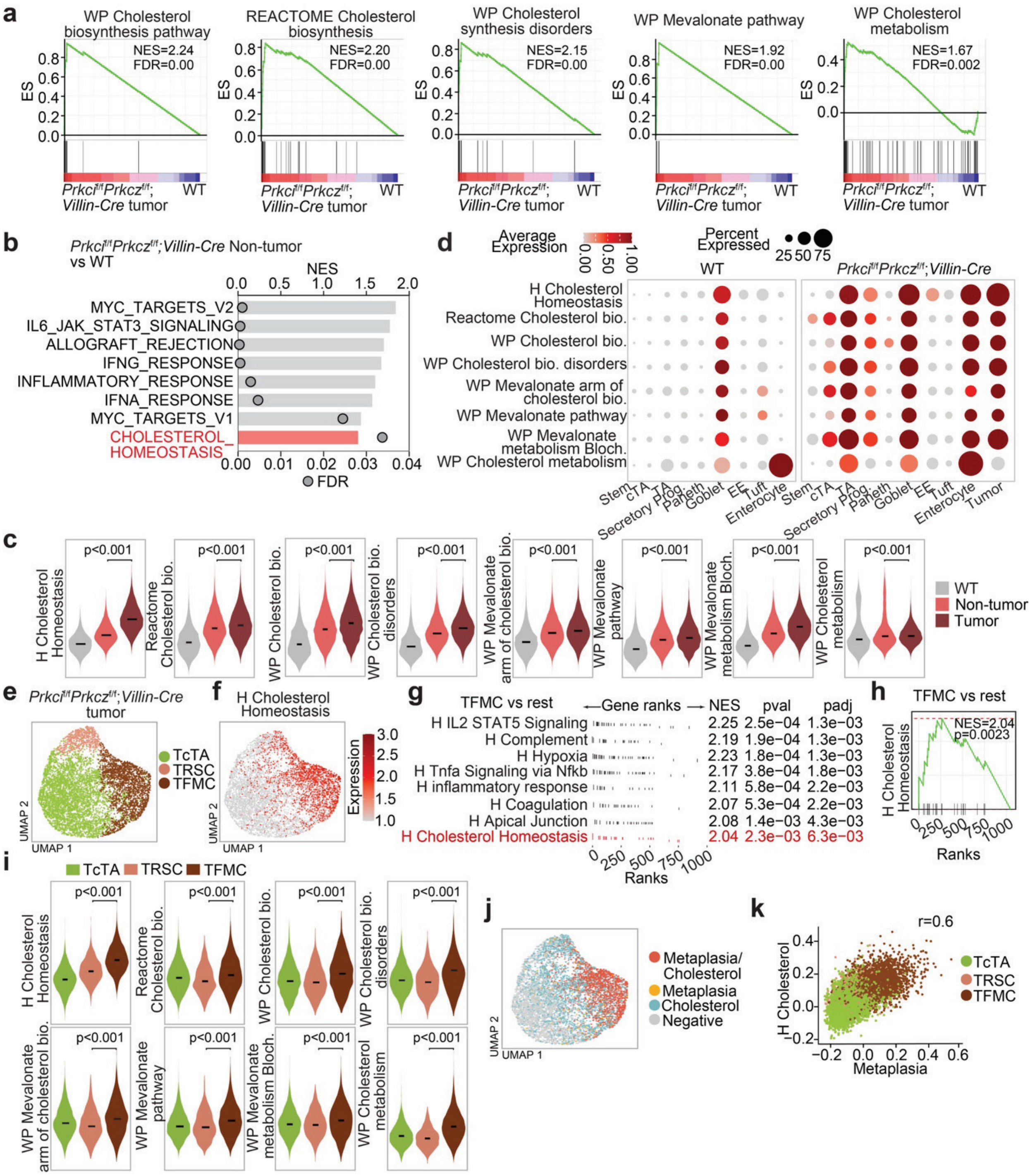

Supplementary Fig. 1. Enhanced cholesterol-related gene expression in aPKC-deficient intestines. a, Gene set enrichment analysis (GSEA) plots of *Prkci*$^{f/f}$*Prkcz*$^{f/f}$*;Villin-Cre* mouse small intestinal tumors (n=3 tumors from three distinct mice) versus WT small intestines (n=3 tissues

from three distinct mice). **b**, GSEA results of the top 8 gene sets in compilation H (MsigDB) for *Prkci*$^{f/f}$*Prkcz*$^{f/f}$*;Villin-Cre* mouse non-tumoral small intestines versus WT small intestines (n=3 tissues from three distinct mice per group). **c-k,** scRNA-seq of mouse small intestinal epithelial cells (n=3 tissues from three distinct WT mice, n=1 tissue from one *Prkci*$^{f/f}$*Prkcz*$^{f/f}$*;Villin-Cre* mouse, and n=5 tumors from two distinct *Prkci*$^{f/f}$*Prkcz*$^{f/f}$*;Villin-Cre* mice). **c**, Violin plots of cholesterol signatures in tissue origins. **d**, Dot plots of cholesterol signatures against cell type in WT and *Prkci*$^{f/f}$*Prkcz*$^{f/f}$*;Villin-Cre* mouse intestinal epithelial cells. **e**, UMAP plot of tumor compartment colored by tumor cell subtypes. **f**, UMAP feature plot of tumor compartment colored by the expression of hallmark cholesterol homeostasis gene set**. g**,**h**, Fast gene set enrichment analysis (FGSEA) comparing tumor fetal metaplastic cells (TFMCs) versus the rest in the tumoral compartment. FGSEA result (**g**) and FGSEA plot (**h**). **i,** Violin plots of indicated gene signatures against tumor cell subtypes in the tumor compartment. **j**, UMAP plot of tumor compartment colored by metaplasia and cholesterol signatures. **k**, Scatter plot and the Pearson correlation coefficient between hallmark cholesterol and metaplasia signature enrichment scores colored by tumor cell subtypes in the tumor compartment. Horizontal lines in violin plots represent median values. Two-tailed, unpaired Student's t-test (**c** and **i**).

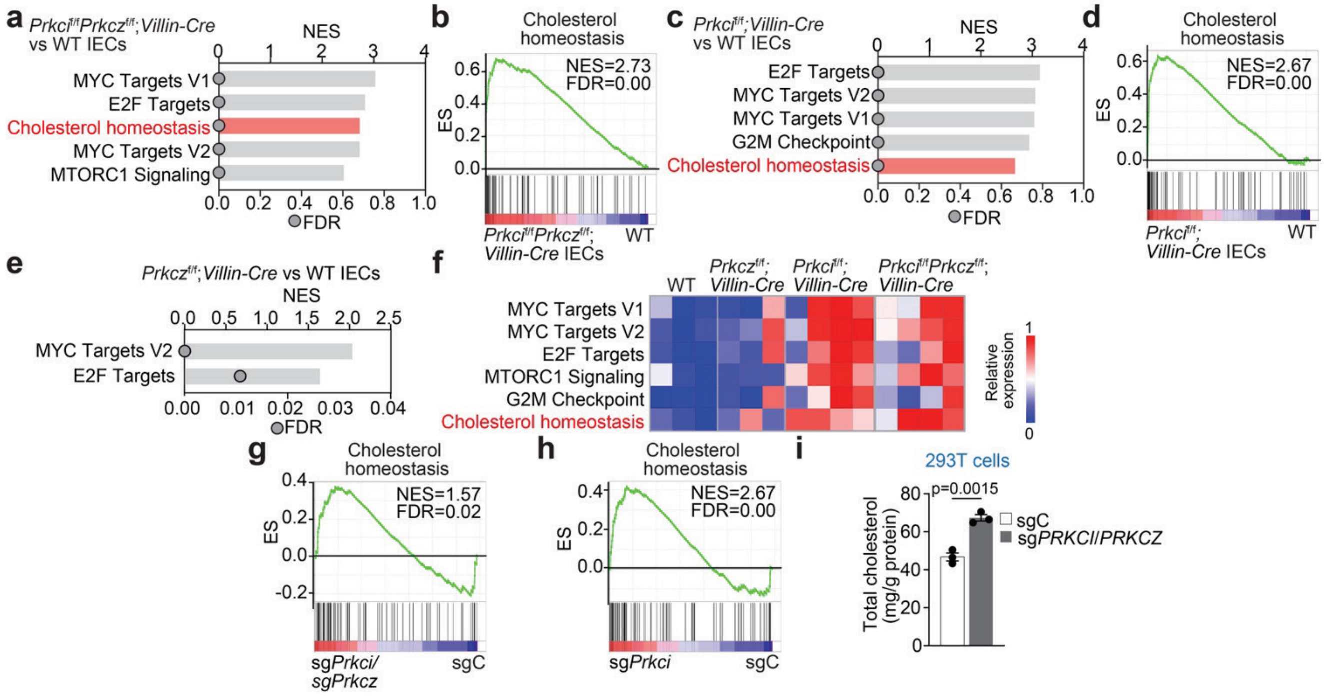

Supplementary Fig. 2: The roles of PKCλ/ι and PKCζ on dysregulated cholesterol metabolism. **a**,**b**, GSEA results of the top 5 gene sets in compilation H for *Prkci*$^{f/f}$*Prkcz*$^{f/f}$*;Villin-Cre* versus WT (**a**) and GSEA plot of *Prkci*$^{f/f}$*Prkcz*$^{f/f}$*;Villin-Cre* versus WT mouse small intestinal epithelial cells (IECs, n=3 biological samples from three distinct WT mice, and n=4 biological samples from four distinct *Prkci*$^{f/f}$*Prkcz*$^{f/f}$*;Villin-Cre* mice) (**b**). **c**,**d**, GSEA results of the top 5 gene sets in compilation H for *Prkci*$^{f/f}$*;Villin-Cre* versus WT (**c**) and GSEA plot of *Prkci*$^{f/f}$*;Villin-Cre;* versus WT mouse small intestinal epithelial cells (IECs, n=3 biological samples three distinct WT, and n=4 biological samples from four distinct *Prkci*$^{f/f}$*;Villin-Cre* mice) (**d**). **e**, GSEA results of gene sets in compilation H (MsigDB) for *Prkcz*$^{f/f}$*;Villin-Cre* versus WT IECs (IECs, n=3 biological samples from three distinct mice per group). **f**, Heat map of Gene Set Variation Analysis (GSVA) results of mouse IECs (n=3 biological samples from three distinct WT mice, n=3 biological samples from three distinct *Prkcz*$^{f/f}$*;Villin-Cre* mice, n=4 biological samples from four distinct *Prkci*$^{f/f}$*;Villin-Cre* mice, and n=4 biological samples from four distinct *Prkci*$^{f/f}$*Prkcz*$^{f/f}$*;Villin-Cre* mice). **g**,**h**, GSEA plots of sg*Prkci*/sg*Prkcz* versus sgC (**g**) and sg*Prkci* versus sgC (**h**) MTOs (n=3 biological replicates per group). **i**, Total cholesterol content in sg*PRKCI*/*PRKCZ* and sgC 293T cells cultured in 1%

LPDS for 24 h (n=3 biological replicates). Data were presented as mean ± SEM. Two-tailed, unpaired Student's t-test (**i**).

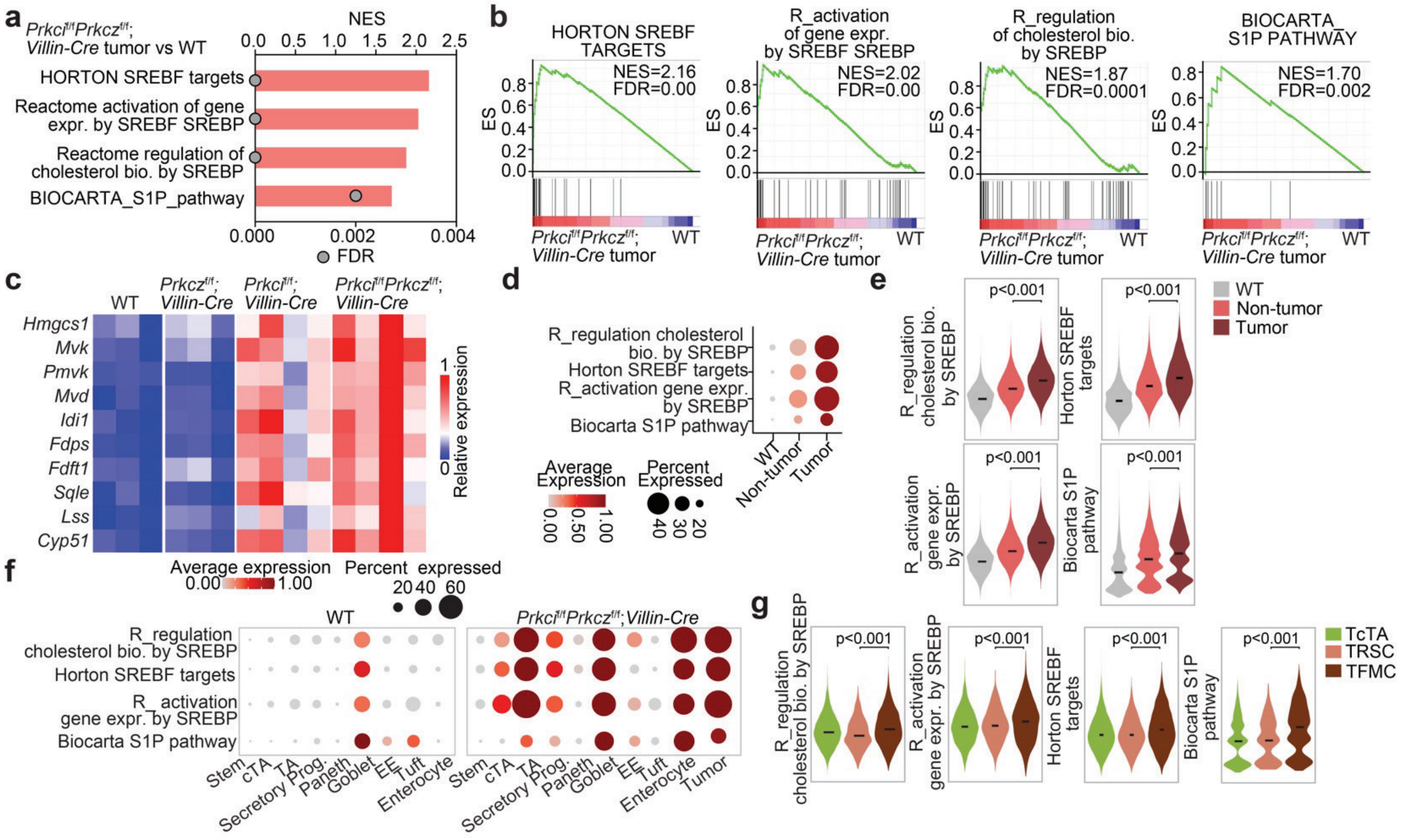

Supplementary Fig. 3. Upregulated SREBP2 activity in aPKC-deficient intestines. a,**b**, GSEA results (**a**) and plots (**b**) of indicated SREBP2 targets signatures for *Prkci*$^{f/f}$*Prkcz*$^{f/f}$*;Villin-Cre* tumors (n=3 tumors from three distinct mice) versus WT small intestines (n=3 tissues from three distinct mice). **c**, Heat map of gene expression from RNAseq data of mouse IECs (n=3 biological samples from three distinct WT mice, n=3 biological samples from three distinct *Prkcz*$^{f/f}$*;Villin-Cre* mice, n=4 biological samples from four distinct *Prkci*$^{f/f}$*;Villin-Cre* mice, and n=4 biological samples from four distinct *Prkci*$^{f/f}$*Prkcz*$^{f/f}$*;Villin-Cre* mice). **d-f**, scRNA-seq of mouse small intestinal epithelial cells (n=3 tissues from three distinct WT mice, n=1 tissue from one *Prkci*$^{f/f}$*Prkcz*$^{f/f}$*;Villin-Cre* mouse, and n=5 tumors from two distinct *Prkci*$^{f/f}$*Prkcz*$^{f/f}$*;Villin-Cre* mice). **d,** Dot plot of indicated SREBP2 target signatures in tissue origins. Violin plots (**e**) and dot plots in tissue of origin by epithelial cell-type (**f**) of SREBP2 target signatures. **g**, Violin plots of SREBP2 target

signatures against tumor cell subtypes in the tumor compartment of *Prkci*$^{f/f}$*Prkcz*$^{f/f}$*;Villin-Cre* mouse small intestine (n=5 tumors from two distinct *Prkci*$^{f/f}$*Prkcz*$^{f/f}$*;Villin-Cre* mice)**.** Horizontal lines represent median values. Two-tailed, unpaired Student's t-test (**e** and **g**).

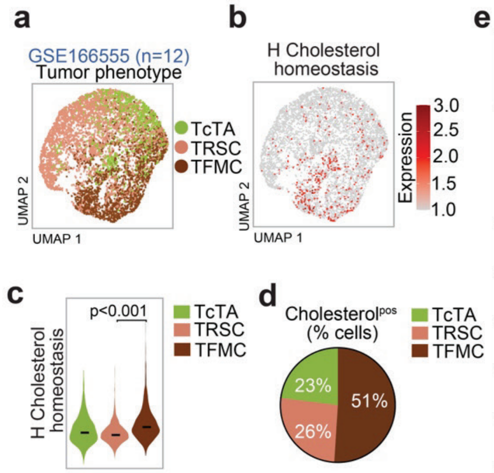

e

Variables	All patients (n=468)	SREBP2-high (n=114)	SREBP2-low (n=354)	p value
Clinical stage				
I	2	1 (50)	1 (50)	0.3940 ns
II	218	52 (23.8)	166 (76.2)	0.8956 ns
III	209	49 (23.4)	160 (76.6)	0.7545 ns
IV	31	9 (29.0)	22 (71.0)	0.5100 ns
aPKCs expression				
Positive	201	37 (18.4)	164 (81.6)	0.0103 *
ANXA10 expression				
Positive	36	16 (44.4)	20 (55.6)	0.0024 **
Primary tumor location				
Proximal	137	45 (32.8)	92 (67.2)	0.0030 **
Lymphatic invasion				
Positive	308	67 (21.7)	241 (78.3)	0.1458 ns
Vascular invasion				
Positive	119	28 (23.5)	91 (76.5)	0.8879 ns
Serrated histology				
Positive	128	53 (41.4)	75 (58.6)	<0.0001 ****

Proximal: Cecum and ascending colon. Values are number (%).

f

Variables	Odds ratio	Odds ratio	95% CI	p value
aPKC expression		0.580	0.354-0.937	0.0277*
Lymphatic invasion		0.664	0.383-1.155	0.1448 ns
Vascular invasion		1.528	0.854-2.715	0.1490 ns
Proximal origin		1.875	1.139-3.072	0.0129*

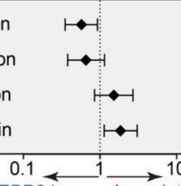

Associated with SREBP2 low — Associated with SREBP2 high

g

Variables	Odds ratio	Odds ratio	95% CI	p value
Serrated CRC		3.076	1.904-4.975	<0.0001****
Lymphatic invasion		0.634	0.372-1.083	0.0942 ns
Vascular invasion		1.482	0.835-2.613	0.1748 ns
Proximal origin		1.902	1.171-3.077	0.0090**

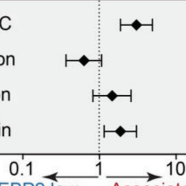

Associated with SREBP2 low — Associated with SREBP2 high

h

Variables	Odds ratio	Odds ratio	95% CI	p value
ANXA10 expression		2.354	1.047-5.127	0.0331*
Lymphatic invasion		0.632	0.370-1.082	0.0926 ns
Vascular invasion		1.497	0.833-2.663	0.1722 ns
Proximal origin		1.779	1.086-2.895	0.0209*

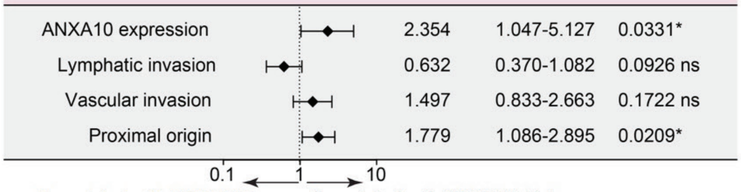

Associated with SREBP2 low — Associated with SREBP2 high

Supplementary Fig. 4. Human relevance of cholesterol metabolism in serrated CRCs. a-d, UMAP plot colored by *Prkci*$^{f/f}$*Prkcz*$^{f/f}$*;Villin-Cre* tumor cell subtypes (**a**), UMAP feature plot colored by the Hallmark cholesterol signature expression (**b**), violin plots dot of indicated gene set split by tumor cell subtypes (**c**), and pie chart of relative distribution of tumor cell subtypes expressing by the Hallmark cholesterol signature (**d**) in tumor epithelial cells (GSE166555, n=12 tumor tissues from 12 patients). Horizontal lines represent median values. **e**, Baseline clinical and pathological characteristics in SREBP2-high and low patients in tissue microarray (TMA). **f-h**, Multivariate logistic regression analyses for the factors associated with high SREBP2 expression. Two-tailed, unpaired Student's t-test (**c**), two-tailed, Mann-Whitney test (**e**).

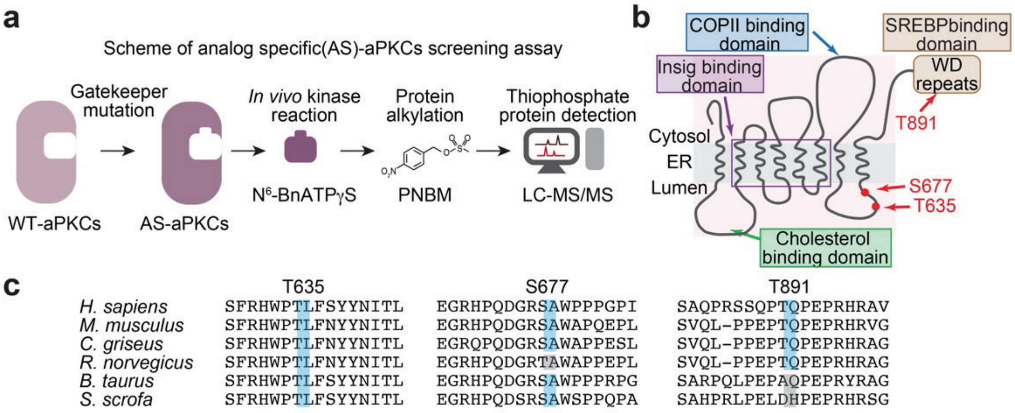

Supplementary Fig. 5: Prediction of PKCλ/ι-mediated SCAP phosphorylation sites. a, Schematic of analog sensitive (AS)-aPKCs screening assay. **b**, Schematic topology of SCAP showing domains and predicted phosphorylation sites by PKCλ/ι. **c**, Amino acid sequence alignment of human SCAP with orthologs in other species.

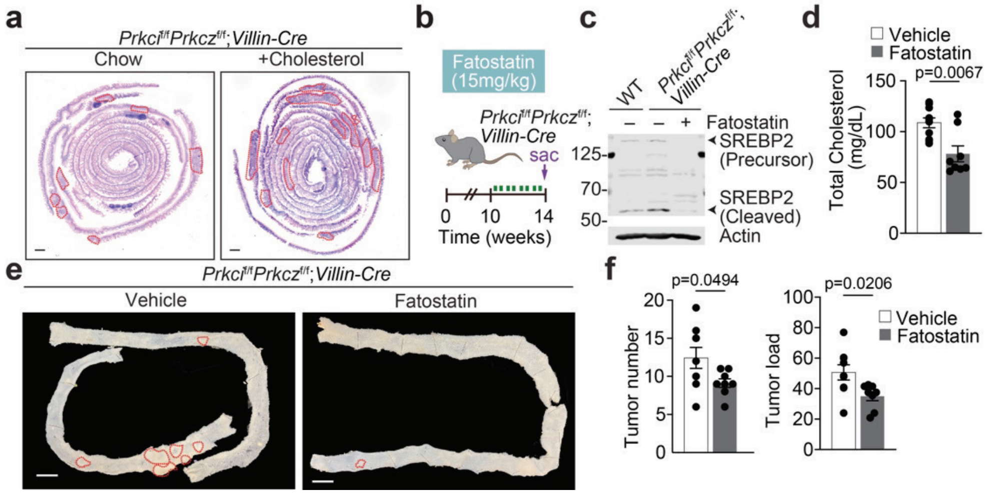

Supplementary Fig. 6. Tumor promotive effect of cholesterol addition in aPKC-deficient tumors. a, Representative H&E staining of small intestine tumors of *Prkci*$^{f/f}$*Prkcz*$^{f/f}$*;Villin-Cre* mice fed with regular chow (n=6 mice per group) or 1.25% cholesterol supplemented diet (n=5 mice per group) for 2 months. **b-f**, *Prkci*$^{f/f}$*Prkcz*$^{f/f}$*;Villin-Cre* mice treated with vehicle (n=9 mice per group) or 15 mg/kg of fatostatin (n=8 mice per group) daily intraperitoneally for 4 weeks. Experimental design (**b**), Immunoblotting of indicated proteins in WT and *Prkci*$^{f/f}$*Prkcz*$^{f/f}$*;Villin-Cre* mice treated with or without fatostatin (**c**), serum total cholesterol levels (**d**), macroscopic images (**e**), total tumor number, and tumor load (**f**). Data were presented as mean ± SEM. Two-tailed, unpaired Student's t-test (**d** and **f**). Scale bars, 1 mm (**a**), 5 mm (**e**).

Supplementary Table 1. List of primers used

Gene Symbol	Forward	Reverse
18s	5’-GTAACCCGTTGAACCCCAT-3’	5’-CCATCCAATCGGTAGTAGCG-3’
Prkci	5’-GCGGGGATATTATGATAACACACTT-3’	5’-TTCCTCATCTATCCATTTCATGGT-3’
Prkcz	5’-TGTGTCCTCACAGATGGAGC-3’	5’-TCCACGGCGGTAGATGGA-3’
Hmgcs1	5’-GGTCTGATCCCCTTTGGTG-3’	5’-TTCAAAGGAAGTGACCCAGG-3’
Hmgcr	5’-TCAGTGGGAACTATTGCACCG-3’	5’-TGGAATGACGGCTTCACAAAC-3’
Ldlr	5’-ATGGAGACCCTCACGGA-3’	5’-TGCTGTTGTTGCCACTG-3’
HMGCS1	5’-GGGCAGGGCATTATTAGGCTAT-3’	5’-TTAGGTTGTCAGCCTCTATGTTGAA-3’
HMGCR	5’-GGGAACCTCGGCCTAATGAA-3’	5’-CACCACGCTCATGAGTTTCCA-3’
LDLR	5’-GTCTTGGCACTGGAACTCGT-3’	5’-CTGGAAATTGCGCTGGAC-3’

Supplementary Table 2. List of guides used

Guides	Source	Identifier
gRNA targeting human *PRKCI,* 5’-TTAAATTATCTTCATGAGCG-3’	Synthego	N/A
gRNA targeting human *PRKCZ,* 5’-CACCTGCAGAGAGCGTACTG-3’	Synthego	N/A

Supplementary Table 3. List of siRNA oligonucleotides used

siRNAs	Source	Identifier
Human *PRCKI* siRNA	Thermo Fisher Scientific	Cat# 110783
Human *TRC8* siRNA	Thermo Fisher Scientific	Cat# 136325
