# Supplementary material for: Enhanced SREBP2-driven cholesterol biosynthesis by PKCλ/ι deficiency in intestinal epithelial cells promotes aggressive serrated tumorigenesis
Source: Nat Commun. 2023 Dec 13;14:8075. doi: 10.1038/s41467-023-43690-5 (PMC10719313; doi:10.1038/s41467-023-43690-5)
Supplement: Supplementary file 3 — Reporting Summary [file 41467_2023_43690_MOESM3_ESM.pdf]

nature portfolio

Corresponding author(s): Jorge Moscat

Last updated by author(s): Oct 23, 2023

Reporting Summary

Nature Portfolio wishes to improve the reproducibility of the work that we publish. This form provides structure for consistency and transparency in reporting. For further information on Nature Portfolio policies, see our Editorial Policies and the Editorial Policy Checklist.

Statistics

For all statistical analyses, confirm that the following items are present in the figure legend, table legend, main text, or Methods section.

n/a	Confirmed	
☐	☒	The exact sample size (*n*) for each experimental group/condition, given as a discrete number and unit of measurement
☐	☒	A statement on whether measurements were taken from distinct samples or whether the same sample was measured repeatedly
☐	☒	The statistical test(s) used AND whether they are one- or two-sided<br>*Only common tests should be described solely by name; describe more complex techniques in the Methods section.*
☐	☒	A description of all covariates tested
☐	☒	A description of any assumptions or corrections, such as tests of normality and adjustment for multiple comparisons
☐	☒	A full description of the statistical parameters including central tendency (e.g. means) or other basic estimates (e.g. regression coefficient) AND variation (e.g. standard deviation) or associated estimates of uncertainty (e.g. confidence intervals)
☐	☒	For null hypothesis testing, the test statistic (e.g. F, t, r) with confidence intervals, effect sizes, degrees of freedom and P value noted<br>*Give P values as exact values whenever suitable.*
☒	☐	For Bayesian analysis, information on the choice of priors and Markov chain Monte Carlo settings
☒	☐	For hierarchical and complex designs, identification of the appropriate level for tests and full reporting of outcomes
☐	☒	Estimates of effect sizes (e.g. Cohen's *d*, Pearson's *r*), indicating how they were calculated

Our web collection on statistics for biologists contains articles on many of the points above.

Software and code

Policy information about availability of computer code

Data collection

Organoids and cell images were collected by EVOS FL Auto Imaging System (Thermo Fisher Scientific) and EVOS M5000 Imaging System (Thermo Fisher Scientific), Histological and immunostaining images were collected by Zeiss LSM 710 NLO Confocal (Microscope Zeiss) and Pannoramic Scanner (3DHistech, Budapest, Hungary). Immunoblotting images were collected by ODYSSEY CLx (LI-COR). qRT-PCR data were collected from Bio-RAD CFX96 Real-Time PCR Detection System.RNA-seq and ATAC-seq data generated in this study was collected by Illumina NextSeq 500 using high outut V2.5 kit.

Data analysis

For RNAseq analysis GenePattern(https://genepattern.broadinstitute.org/gp/pages/index.jsf) was used to collapse gene matrix files (CollapseDataset module) or to assess the statistical significance of differential gene expression (DESeq2). Genes were sorted by log2 FC>0.5 and adj>0.05. Gene Set Enrichment Analysis (GSEA) was performed using GSEA 4.0 software (http://www.broadinstitute.org/gsea/index.jsp) with 1000 gene-set permutations using the gene-ranking metric t-test. For ATACseq analysis, FASTQ files from ATAC-seq reads were aligned to UCSC mm10 with Bowtie2 (bowtie2 –very-sensitive -x mm10 -1 FILE_merged_R1.fastq –2 FILE_merged_R2.fastq -X 1000 -p 12 | samtools view -u - | samtools sort - > FILE.bam). Peak calling was performed with MACS2 with a threshold of q < 0.05. Peaks were annotated with ChipSeeker R package. For scRNAseq analysis Raw sequence reads were quality-checked using FastQC software. The Cell Ranger version 3.0 software suite from 10X Genomics (https://support.10xgenomics.com/single-cell-gene-expression/software/downloads/latest) was used to process, align and summarize unique molecular identifier (UMI) counts against the mouse mm10 assembly reference genome analysis set, obtained from the University of California Santa Cruz (UCSC). Raw, unfiltered count matrices were imported into R for further processing. Raw UMI count matrices were filtered using the Seurat v 3.0 R package to remove: barcodes with very low (less than 200, empty wells) and very high (more than 4000, probably doublets) total UMI counts; matrices for which a high percentage of UMIs originated from mitochondrial features (more than 12%); and matrices for which fewer than 250 genes were expressed. Subsequently, the data were normalized using the SCTransform function, regressing out the following variables: total number of UMIs per cell and percentage of mitochondrial UMIs. Following

normalization, the principal components were computed. The top principal components were identified using the ElbowPlot function and used for the UMAP dimensionality reduction. For clustering, we used Seurat in combination with Harmony software to correct the potential effects of technical differences between sequencing batches.Raw mass spectrometry data were processed using the MaxQuant software package and Andromeda search engine (version 1.5.5.1). Additional software used: Microsoft Excel (version:16.59)Graphpad Prism 9 (Graphpad), QuPath v.0.1.3 (Queen's University, Belfast, Northern Ireland),ImageJ (NIH), BioRender (BioRender), Zen blue (Zeiss) RStudio (1.1.456) R Core Team, R R Core Team GSVA; (version 1.26.0 Bioconductor),Morpheus, (Broad Institute),GenePattern Broad Institute, Lexogen QuantSeq DE 1.3.0 BlueBee Cloud , fgsea (v1.18.0, R package), PhenoChart Whole Slide Viewer AKOYA BIOSCIENCES and CaseViewer 3DHistech, Budapest, Hungary

For manuscripts utilizing custom algorithms or software that are central to the research but not yet described in published literature, software must be made available to editors and reviewers. We strongly encourage code deposition in a community repository (e.g. GitHub). See the Nature Portfolio guidelines for submitting code & software for further information.

Data

Policy information about availability of data

All manuscripts must include a data availability statement. This statement should provide the following information, where applicable:

- Accession codes, unique identifiers, or web links for publicly available datasets
- A description of any restrictions on data availability
- For clinical datasets or third party data, please ensure that the statement adheres to our policy

The data generated in this study are available in the GEO database under the accession numbers GSE239917 [https://www.ncbi.nlm.nih.gov/geo/query/acc.cgi?acc=GSE239917] for bulk RNA-seq, GSE236848 for scRNA-seq data and GSE236850 for ATAC-seq data (Superseries GSE236851,[https://www.ncbi.nlm.nih.gov/geo/query/acc.cgi?acc=GSE236851]) . Data for TCGA-COREAD was accessed through cBioportal (https://www.cbioportal.org). Raw gene expression data of CRC patient dataset (GSE79462 [https://www.ncbi.nlm.nih.gov/geo/query/acc.cgi?acc=GSE79462], GSE76987 [https://www.ncbi.nlm.nih.gov/geo/query/acc.cgi?acc=GSE76987] and GSE4045 [https://www.ncbi.nlm.nih.gov/geo/query/acc.cgi?acc=GSE4045]) were directly accessed through the GEO website (NCBI).Raw gene expression (count) data and metadata of single cell transcriptomic dataset of human CRCs were directly accessed through the GEO website (GSE132465 [https://www.ncbi.nlm.nih.gov/geo/query/acc.cgi?acc=GSE132465] and GSE166555 [https://www.ncbi.nlm.nih.gov/geo/query/acc.cgi acc=GSE166555], and converted into Seurat object Raw gene expression data from Mouse tumor organoids were directly accessed through the GEO website (GSE207776 [https://www.ncbi.nlm.nih.gov/geo/query/acc.cgi]). Raw gene expression data from DKO mice from normal and tumor epithelium were directly accessed through the GEO website (GSE109289 [https://www.ncbi.nlm.nih.gov/geo/query/acc.cgi?acc=GSE109289]). ATAC seq from small intestinal epithelial cells were directly accessed the GEO website (GSE180223 [https://www.ncbi.nlm.nih.gov/geo/query/acc.cgi?acc=GSE180223]). Published single cell transcriptome data of DKO tumor was downloaded from GEO website GSE207780 [https:// www.ncbi.nlm.nih.gov/geo/query/acc.cgi?acc=GSE207780]), and converted into Seurat object.

Tokens for access of reviewers:

To review GEO accession GSE239917:
Go to https://www.ncbi.nlm.nih.gov/geo/query/acc.cgi?acc=GSE239917
Enter token klovyooczduvpun into the box

To review GEO accession GSE236851:
Go to https://www.ncbi.nlm.nih.gov/geo/query/acc.cgi?acc=GSE236851
Enter token kleroewkjdqvryv into the box

Research involving human participants, their data, or biological material

Policy information about studies with human participants or human data. See also policy information about sex, gender (identity/presentation), and sexual orientation and race, ethnicity and racism.

Reporting on sex and gender	TMAs of surgically resected human CRC samples were obtained from males (n = 194, age median:66.1 years) and females (n = 189, age median:67.8 years) at Osaka Metropolitan University Hospital and human CRC samples from males (n = 50, age median:70.0 years) and females (n = 50, age median:69.9 years) at Santa Lucía General University Hospital (HGUSL), Spain.
Reporting on race, ethnicity, or other socially relevant groupings	This study do not involve any race, ethnicity or socially relevant category considered for the analysis
Population characteristics	TMAs of surgically resected from 390 individuals with CRC were obtained at Osaka Metropolitan University Hospital, from males (n = 194, age median:66.1 years) and females (n = 189, age median:67.8 years) from those n=381 were as followed: stage 2: n = 192, stage 3: n = 163, and stage 4: n=26. TMAs of surgically resected from males (n = 50, age median:70.0 years) and females (n = 50, age median:69.9 years) with CRC were obtained at Santa Lucía General University Hospital, from those n=95 were as follow: pT1: n=2, pT2:n=5, pT3: n=60, pT4:n=28. CRC organoids were generated from adult patients with a disease diagnoses using either surgical resection or endoscopic biopsy as starting material and were de-identified to the research team.
Recruitment	For collecting TMA samples, patients with CRCs who underwent surgical resection in the period between January 2007 and December 2012 at Osaka Metropolitan University Hospital and between January 2017 and December 2020 at Santa Lucía

	General University Hospital (HGUSL), Spain were recruited. Patients were recruited for organoid generation in 2021 at Weill Cornell presbyterian hospital with informed consent under the approval of the IRB Committee of Weill Cornell Medicine. Patients were not specifically recruited for this study.
Ethics oversight	This study was approved by the Osaka City University Ethics Committee and the HSUGL ethical board and written informed consent was obtained from the patients. The study was approved by the IRB Committee of Weill Cornell Medicine

Note that full information on the approval of the study protocol must also be provided in the manuscript.

Field-specific reporting

Please select the one below that is the best fit for your research. If you are not sure, read the appropriate sections before making your selection.

☒ Life sciences ☐ Behavioural & social sciences ☐ Ecological, evolutionary & environmental sciences

For a reference copy of the document with all sections, see nature.com/documents/nr-reporting-summary-flat.pdf

Life sciences study design

All studies must disclose on these points even when the disclosure is negative.

Sample size	Sample size was determined based on animal availability and accepted sample sizes for these types of experiments. For in vitro experiments , previously published results, complexity, and past experience were used to determine the sample size. Reproducibility between biological replicates and independent experiments as well as the magnitude and consistency of measurable differences between groups was also considered.
Data exclusions	We have not excluded any data.
Replication	Animal experiments were independently performed for at least two times with more than three replicated. In vitro data have been performed independently for at least three times with three replicates
Randomization	Sex- (male and female) and age- (4-10 weeks old) matched animals were randomly allocated from each genotype into experimental groups. For in vitro studies, cells same batch of cells were plated and the wells were grouped randomly for each experimental parameter
Blinding	An identification code was assigned to each animal and the investigators were not blinded to group allocation at the time of data collection and analysis. Blinding was not possible because the diets and treatments were different and it was technically/practically to do so.

Reporting for specific materials, systems and methods

We require information from authors about some types of materials, experimental systems and methods used in many studies. Here, indicate whether each material, system or method listed is relevant to your study. If you are not sure if a list item applies to your research, read the appropriate section before selecting a response.

Materials & experimental systems

n/a	Involved in the study	
☐	☒	Antibodies
☐	☒	Eukaryotic cell lines
☒	☐	Palaeontology and archaeology
☐	☒	Animals and other organisms
☒	☐	Clinical data
☒	☐	Dual use research of concern
☒	☐	Plants

Methods

n/a	Involved in the study	
☒	☐	ChIP-seq
☒	☐	Flow cytometry
☒	☐	MRI-based neuroimaging

Antibodies

Antibodies used	The antibodies used in this study are described below. All antibodies were validated by manufacter. Mouse anti-β-actin,Sigma-Aldrich,"Cat# A1978, RRID: AB_476692", Rabbit anti-aPKCs,Abcam,Cat# ab59364; RRID: AB_944858, Mouse anti-PKCλ/ι,BD Biosciences,"Cat# 610208, RRID: AB_397607", Mouse anti-FLAG,Sigma-Aldrich,"Cat# F3165, RRID: AB_259529", Rabbit anti-HA-Tag,Cell Signaling Technology,"Cat# 3724, RRID: AB_1549585", Rabbit anti-SREBP2,Abcam,"Cat#ab30682, RRID: AB_779079", Rabbit anti-GFP,Cell Signaling Technology,"Cat#2956, RRID: AB_1196615", Rabbit anti-GFP,Abcam,"Cat#ab290, RRID: AB_303395",Rabbit anti-Thiophosphate ester,Abcam,"Cat# ab92570, RRID: AB_10562142", Mouse anti-SCAP,Santa Cruz Biotechnology,"Cat# sc-13553, RRID: AB_628237", Rabbit anti-SCAP,Thermo Fisher Scientific,"Cat# PA5-28982, RRID: AB_2546458", Mouse anti-Ubiquitin,Santa Cruz Biotechnology,"Cat# sc-8017, RRID: AB_2762364", Rabbit anti-Ki67,Cell Signaling Technology,"Cat#12202, RRID: AB_2620142", Rabbit anti-ANXA10,Abcam,"Cat# ab213656, RRID: AB_2921231",Rabbit anti-Cleaved Caspase-3,Cell Signaling Technology,"Cat# 9664, RRID: AB_2070042", Mouse anti-TRC8,Santa Cruz Biotechnology,"Cat# sc-390347, RRID: AB_3073784", Normal Rabbit IgG,Cell Signaling Technology,"Cat# 2729, RRID: AB_1031062", Normal mouse IgG,Santa Cruz Biotechnology,"Cat# sc-2025, RRID: AB_737182", "Goat anti-Mouse IgG1, secondary, HRP",Thermo Fisher Scientific,"Cat# PA1-74421, RRID: AB_10988195", "Goat anti-

	Rabbit IgG, secondary, HRP",Thermo Fisher Scientific,"Cat# 31461, RRID: AB_228347","Goat anti-Mouse IgG1, secondary, Alexa Fluor 488",Thermo Fisher Scientific,"Cat# A21121, RRID: AB_2535764","Donkey anti-Rat IgG, secondary, Alexa Fluor 488",Thermo Fisher Scientific,"Cat# A21208, RRID: AB_2535794","Donkey anti-Rabbit IgG, secondary, Alexa Fluor 568",Thermo Fisher Scientific,"Cat# A10042, RRID: AB_2534017","Goat anti-Rabbit IgG, secondary, IRDye 800",LI-COR Biosciences,"Cat# 926-32211, RRID: AB_621843", "Goat anti-Mouse IgG1, secondary, IRDye 800",LI-COR Biosciences,"Cat# 926-32350, RRID: AB_2782997", "Goat anti-Mouse IgG, secondary, IRDye 800",LI-COR Biosciences,"Cat# 926-32210, RRID: AB_621842".
Validation	Antibodies were validated by the manufacturer as stated on the manufacturers website. Manufacturer's validation statements are described on the following websites: Mouse anti-β-Actin (A1978; Sigma-Aldrich) [https://www.sigmaaldrich.com/US/en/product/sigma/a1978], Rabbit anti-aPKCs (ab59364; Abcam) [https://www.abcam.com/products/primary-antibodies/pkc-zeta-antibody-ab59364.html], Mouse anti-PKCλ/ι (610208; BD Biosciences) [https://www.bdbiosciences.com/en-us/products/reagents/microscopy-imaging-reagents/immunofluorescence-reagents/purified-mouse-anti-pkc.610208], Mouse anti-FLAG (F3165; Sigma-Aldrich) [https://www.sigmaaldrich.com/US/en/product/sigma/f3165], Rabbit anti-HA-Tag (3724; Cell Signaling Technology) [https://www.cellsignal.com/products/primary-antibodies/ha-tag-c29f4-rabbit-mab/3724], Rabbit anti-SREBP2 (ab30682; Abcam) [https://www.abcam.com/products/primary-antibodies/srebp2-antibody-ab30682.html], Rabbit anti-GFP (2956; Cell Signaling Technology) [https://www.cellsignal.com/products/primary-antibodies/gfp-d5-1-rabbit-mab/2956], Rabbit anti-GFP (ab290; Abcam) [https://www.abcam.com/products/primary-antibodies/gfp-antibody-ab290.html], Rabbit anti-Thiophosphate ester (ab92570; Abcam) [https://www.abcam.com/products/primary-antibodies/thiophosphate-ester-antibody-51-8-ab92570.html], Mouse anti-SCAP (sc-13553; Santa Cruz Biotechnology) [https://www.scbt.com/p/scap-antibody-9d5], Rabbit anti-SCAP (PA5-28982; Thermo Fisher Scientific) [https://www.thermofisher.com/order/genome-database/generatePdf?productName=SCAP&assayType=PRANT&detailed=true&productId=PA5-28982], Mouse anti-Ubiquitin (sc-8017; Santa Cruz Biotechnology) [https://www.scbt.com/p/ubiquitin-antibody-p4d1], Rabbit anti-Ki67 (12202; Cell Signaling Technology) [https://www.cellsignal.com/products/primary-antibodies/ki-67-d3b5-rabbit-mab-mouse-preferred-ihc-formulated/12202], Rabbit anti-ANXA10 (ab213656; Abcam) [https://www.abcam.com/products/primary-antibodies/annexin-a10anxa10-antibody-epr19507-ab213656.html], Rabbit anti-Cleaved Caspase-3 (9664; Cell Signaling Technology) [https://www.cellsignal.com/products/primary-antibodies/cleaved-caspase-3-asp175-5a1e-rabbit-mab/9664], Mouse anti-TRC8 (sc-390347; Santa Cruz Biotechnology) [https://www.scbt.com/p/trc8-antibody-h-9], Normal Rabbit IgG (2729; Cell Signaling Technology) [https://www.cellsignal.com/products/primary-antibodies/normal-rabbit-igg/2729], Normal Mouse IgG (sc-2025; Santa Cruz Biotechnology) [https://www.scbt.com/p/normal-mouse-igg], Goat anti-Mouse IgG1, secondary, HRP (PA1-74421; Thermo Fisher Scientific) [https://www.thermofisher.com/antibody/product/Goat-anti-Mouse-IgG1-Secondary-Antibody-Polyclonal/PA1-74421], Goat anti-Rabbit IgG, secondary, HRP (31461; Thermo Fisher Scientific) [https://www.thermofisher.com/antibody/product/Goat-anti-Rabbit-IgG-F-ab-2-Secondary-Antibody-Polyclonal/31461], Goat anti-Mouse IgG1, secondary, Alexa Fluor 488 (A21121; Thermo Fisher Scientific) [https://www.thermofisher.com/antibody/product/Goat-anti-Mouse-IgG1-Cross-Adsorbed-Secondary-Antibody-Polyclonal/A-21121], Donkey anti-Rat IgG, secondary, Alexa Fluor 488 (A21208; Thermo Fisher Scientific) [https://www.thermofisher.com/antibody/product/Donkey-anti-Rat-IgG-H-L-Highly-Cross-Adsorbed-Secondary-Antibody-Polyclonal/A-21208], Donkey anti-Rabbit IgG, secondary, Alexa Fluor 568 (A10042; Thermo Fisher Scientific) [https://www.thermofisher.com/antibody/product/Donkey-anti-Rabbit-IgG-H-L-Highly-Cross-Adsorbed-Secondary-Antibody-Polyclonal/A10042], Goat anti-Rabbit IgG, secondary, IRDye 800 (926-32211; LI-COR Biosciences) [https://www.licor.com/bio/reagents/irdye-800cw-goat-anti-rabbit-igg-secondary-antibody], Goat anti-Mouse IgG1, secondary, IRDye 800 (926-32350; LI-COR Biosciences) [https://www.licor.com/bio/reagents/irdye-800cw-goat-anti-mouse-igg1-specific-secondary-antibody], Goat anti-Mouse IgG, secondary, IRDye 800 (926-32210; LI-COR Biosciences) [https://www.licor.com/bio/reagents/irdye-800cw-goat-anti-mouse-igg-secondary-antibody].

Eukaryotic cell lines

Policy information about cell lines and Sex and Gender in Research

Cell line source(s)	Human HEK293T (sex:female) was purchased from ATCC (CRL-3216)<br>Human HCT116 (sex:male) was purchased from ATCC (CCL-247)<br>Mouse L Wnt-3A (sex:male) was purchased from ATCC (CRL-2647)<br>Mouse tumor organoids (MTO, sex:male); kindly provided by Dr. Battle's laboratory<br>Human patient-derived organoids (PDO, sex: male); this study
Authentication	None of the cell lines was authenticated.
Mycoplasma contamination	Cell lines were tested routinely and were all negative for mycoplasma contamination.
Commonly misidentified lines (See ICLAC register)	None of the misidentified lines were used in this study

Animals and other research organisms

Policy information about studies involving animals; ARRIVE guidelines recommended for reporting animal research, and Sex and Gender in Research

Laboratory animals	Generation of Villin-Cre-Prkcz-/- Llado V, et al, Cell Reports, 2015), Villin-Cre-Prkci-/- (Nakanishi Y, et al, Cell Reports, 2016) Villin-Cre-Prkcz-/- Prkci-/- (Nakanishi Y, et al, Immunity, 2018) and Villin-creERT2-Prkcz-/-Prkci-/- (Martinez-Ordonez et al, Cancer Cell, 2023) mice were previously described. Villin-Cre, Villin-creERT2 and C57BL/6 mice were purchased from The Jackson Laboratory (stock numbers 004586,020282 and 000664 respectively). All mouse strains were generated in a C57BL/6 background. 8-12 week-old mice were used in all experiments. 4-6 week-old NOD.Cg-Prkdcscid Il2rgtm1Wjl/SzJ (NSG) mice were purchased from The Jackson Laboratory (stock number 005557).<br>The mice were maintained in 14 h light / 10 h dark cycle, and the housing temperature and humidity were 24 C and 50%, respectively.

Wild animals	This study do not involve wild animals
Reporting on sex	Both sexes were used in this study. Animals were sex-matched and randomly assigned. Cholesterol-related phenotypes, tumor growth, and response to treatment were comparable between the sexes. Overall numbers are as follows. Villin-Cre-Prkcz-/- Prkci-/- (male: n=20, female: n=40), C57BL/6 (male: n=10, female: n=10), NSG (male: n=2, female: n=2).
Field-collected samples	The study did not involve any field-collected samples
Ethics oversight	Animal handling and experimental procedures conformed to institutional guidelines and were approved by the Sanford-Burnham-Prebys Medical Discovery Institute Institutional Animal Care and Use Committee, and by the Weill Cornell Medicine Institutional Animal Care and Use Committee.

Note that full information on the approval of the study protocol must also be provided in the manuscript.
